# Nature Inspired Engineering: Biomimetic Sensors

**DOI:** 10.3390/s25154807

**Published:** 2025-08-05

**Authors:** Kiyoshi Toko, Xiao Wu

**Affiliations:** 1 Division of Taste Sensor, Research and Development Center for Five-Sense Devices, Kyushu University, Fukuoka 819-0395, Japan; 2 Department of Information Electronics, Faculty of Engineering, Fukuoka Institute of Technology, 3-30-1 Wajiro-higashi, Higashi-ku, Fukuoka 811-0295, Japan; xiao@fit.ac.jp

This Special Issue of *Sensors* highlights recent advances in biomimetic sensor research, encompassing both fundamental concepts and practical implementations. Nature offers a wealth of inspiration for sensor design. Notably, electronic tongues and electronic noses represent analytical systems often composed of arrays of semi-selective chemical or biological sensors, coupled with multivariate data analysis techniques. Because their underlying design is modeled after biological sensory mechanisms, these devices are referred to as biomimetic sensors. Over the last thirty years, they have been utilized in numerous applications, such as sample classification based on intended use, quantitative evaluation of taste, and flavor profiling. Biomimetic sensors emulate human sensory systems to recognize a variety of external stimuli. With superior sensitivity, selectivity, and precision compared to natural human senses, they contribute to exploring the unknown and enhancing everyday life.

A total of ten articles, comprising both literature reviews and original research articles, are presented, covering diverse topics such as biomimetic sensing materials, bioinspired sensors, electronic tongues, electronic noses, taste sensors, odor sensing arrays, olfaction proteins, olfactory receptors, biological tissues, cell sensors, biomedical sensors, data analysis, MEMS, environmental analysis, biomedical applications, gas sensors, multi-array sensors, machine learning, AI (artificial intelligence), food sensors, image sensors, and wearable sensors.

Wang et al. (Contribution 1) introduced a multifunctional imaging sensor inspired by the mantis shrimp’s exceptional visual system, capable of simultaneously capturing spectral and polarization information over a broad wavelength range. Their findings offer significant potential for the advancement of high-performance, versatile optical sensors, opening up new avenues in optical information acquisition. Chen et al. (Contribution 2) designed an electrochemical immunosensor with antifouling properties for label-free detection of the biomarker carbohydrate antigen 19-9 (CA19-9). In this system, bovine serum albumin (BSA) and graphene were cross-linked using glutaraldehyde to construct a three-dimensional conductive porous structure on the electrode surface. The experimental results demonstrated that the sensor exhibits excellent performance in detecting CA19-9, indicating its strong potential for clinical diagnostic applications.

Watanabe et al. (Contribution 3) conducted a fundamental study aimed at enhancing taste sensors based on lipid polymer membranes, focusing on reducing sensitivity to anions such as nitrate (NO_3_^−^) ions, which are present in vegetables but lack taste. Their findings support the development of sensor membranes with selective responses to various anions, paving the way for taste sensors that can minimize interference from tasteless anionic species. Iitani et al. (Contribution 4) developed biosensors (bio-sniffers) for both liquid and gas phases, aiming to enable simple and continuous detection of *trans*-2-nonenal vapor, a key compound associated with age-related body odor. They compared two nicotinamide adenine dinucleotide (phosphate)-dependent enzymes—aldehyde dehydrogenase and enone reductase 1. The results revealed age-related variations in signal responses, indicating the feasibility of detecting *trans*-2-nonenal vapor. Faricha et al. (Contribution 5) designed a miniaturized sensor aimed at achieving rapid response, easy chip integration, and the detection of lower concentrations of target compounds. To enhance sensitivity toward butanol isomer gases, atomic gold clusters (Au_2_) were applied as a catalyst on a platinum/polyaniline (Pt/PANI) working electrode. As a result, the sensor demonstrated excellent current density and a strong linear relationship with concentration.

Jing et al. (Contribution 6) created a highly sensitive sensor incorporating lipid/polymer membranes embedded with a Na^+^ ionophore, designed to detect the saltiness enhancement effect caused by the addition of specific compounds to sodium chloride. The sensor successfully quantified this enhancement, and the findings are expected to contribute to the development of reduced-sodium foods that promote healthier dietary habits. Yu et al. (Contribution 7) provided a comprehensive review of recent progress in bionic sensors for biometric data acquisition and monitoring, highlighting four key technological areas: bioelectric signal sensors, biomarker sensors, biomechanical sensors, and multimodal integrated sensors. The successful development of bionic sensors, with wide-ranging applications in life sciences, smart healthcare, and medical diagnostics, relies on close interdisciplinary cooperation among engineers, scientists, and medical professionals.

Uchida (Contribution 8) provides a review of taste sensors with a focus on evaluating the bitterness of oral medications. The paper also describes a novel taste sensor featuring lipid/polymer membranes modified with dihydroxybenzoic acids, designed based on the principle of allostery. Finally, future directions for taste sensor development are discussed. The COVID-19 pandemic has had significant social and economic impacts worldwide. Yasuura et al. (Contribution 9) reviewed a range of virus detection methods and highlighted three advanced techniques—bead-based assays, digital assays, and pore-based sensing—that enhance the speed and sensitivity of viral detection. Kuroda et al. (Contribution 10) reviewed the current progress and future outlook of human olfactory digital transformation (DX), focusing on odor sensors that incorporate olfactory receptors (ORs)—the molecules responsible for human smell perception—as sensing elements (i.e., human OR sensors). A major challenge in this field is achieving real-time odor reproduction, which could revolutionize the industry if integrated with video devices capable of delivering targeted scents on demand.

As demonstrated, many nature-inspired sensors and devices have been reported, underscoring the rich inspiration drawn from nature in sensor design. This collection of papers constitutes a vital resource for professionals, researchers, and engineers working in this field. The editors would like to express their deepest appreciation to all contributing authors for their outstanding research, as well as to the *Sensors* editorial board for their assistance in promoting these valuable studies.

